# Predicting Chemical Composition and Apparent Total Tract Digestibility on Freeze-Dried Not Ground Faeces Using Near-Infrared Spectroscopy in Pigs

**DOI:** 10.3390/ani13132090

**Published:** 2023-06-24

**Authors:** Jordi Camp Montoro, David Solà-Oriol, Ramon Muns, Josep Gasa, Núria Llanes, Edgar Garcia Manzanilla

**Affiliations:** 1 Pig Development Department, Animal and Grassland Research and Innovation Centre, Teagasc, Moorepark, Co. Cork, P61 C996 Fermoy, Ireland; egmanzanilla@gmail.com; 2 Animal Nutrition and Welfare Service, Department of Animal and Food Sciences, Universitat Autònoma de Barcelona, 08193 Bellaterra, Spain; david.sola@uab.cat (D.S.-O.); josep.gasa@uab.cat (J.G.); 3 Agri-Food and Biosciences Institute, Large Park, Hillsborough, Co Down, Northern Ireland BT 26 6DR, UK; ramon.muns@afbini.gov.uk; 4 Cooperativa d’Ivars d’Urgell SCCL, Ivars d’Urgell, 25260 Lleida, Spain; nllanes@coopivars.coop; 5 UCD Veterinary Sciences Centre, University College Dublin, Belfield, Dublin 4, D04 V1W8 Dublin, Ireland

**Keywords:** diet, digestibility, faeces, NIRS, nutrition, pig, swine

## Abstract

**Simple Summary:**

Near-infrared reflectance spectroscopy (NIRS) is widely used to predict the nutritional value of raw materials and complete feeds in feed mills. Recently, faecal NIRS was proposed as a fast and cheap method to predict nutrient digestibility to partially replace in vivo digestibility trials. Previous studies used freeze-dried ground (FDG) faeces via NIRS, but the use of not ground (FDNG) faeces could save time and workload. The objective of the present study was to compare the results obtained with NIRS using faecal samples in two forms, FDNG and FDG, to predict faecal chemical composition and apparent total tract digestibility (ATTD) coefficients. Faecal samples were collected from grower-finisher pigs at pen level, freeze-dried, and analysed via NIRS as FDNG and FDG faeces. NIRS calibrations were developed and successfully predicted faecal chemical components and ATTD coefficients of nutrients for both FDNG and FDG faeces. Thus, faecal NIRS is a potential tool to evaluate faeces’ chemical components and ATTD coefficients of nutrients, and those are successfully predicted using FDNG faeces, saving analysis time and workload.

**Abstract:**

The present study aimed to compare NIRS results using freeze-dried ground or not ground (FDG or FDNG) faeces to predict faecal chemical composition and apparent total tract digestibility (ATTD) coefficients. Two different batches of pigs were used (*n* = 20 mixed sex pens/batch; 11 pigs/pen; Duroc × (Large White × Landrace)). The first batch of pigs (B1; 50.1 ± 3.44 kg body weight (BW)) was used at 13 wks of age and the second batch (B2; 87.0 ± 4.10 kg BW) was used at 18 wks of age. For both B1 and B2, pens were assigned to five diets formulated to obtain a control [10.03 MJ of net energy (NE), 160.0 g of crude protein (CP), and 9.5 g of standardized ileal digestive (SID) lysine (Lys) per kg of feed], low protein (132.0 g CP and 7.5 g SID Lys), high protein (188.0 g CP and 11.5 g SID Lys), low energy (9.61 MJ NE/kg), and high energy (10.45 MJ NE/kg) diets. After a 10-day adaptation period, one faecal sample was collected daily from each pen floor during 6 days in both B1 and B2 (*n* = 120/batch). Faecal samples were freeze-dried and analysed via NIRS as FDNG and FDG faeces. Dry matter (DM), organic matter (OM), CP, gross energy (GE), fat, and ATTD coefficients were analysed/calculated. The NIRS calibrations were evaluated by cross-validation, splitting the data in four random groups, or using the leave-one-out method. For both FDNG and FDG faeces, coefficients of determination for calibration (R^2^_cv_) and residual predictive deviation (RPD) values were: close to 0.9 and 3 for DM and CP, 0.7–0.8 and ≥2 for OM and GE, 0.6 and <2 for fat, and 0.54–0.75 and ≤2 for ATTD coefficients, respectively. CP was better predicted using FDG faeces (*p* < 0.05), while DM and OM ATTD were better predicted using FDNG faeces (*p* < 0.05). In conclusion, NIRS successfully predicts faeces’ chemical components and ATTD coefficients of nutrients using FDNG or FDG faeces.

## 1. Introduction

Diet optimisation is currently a time-consuming process that includes ingredient analysis, digestibility determination, and on-farm feed efficiency measurements. Describing the nutritional value of ingredients for livestock should be performed in vivo using chemical analysis of the feed and prediction equations for each type of animal [1]. Nonetheless, feed efficiency will vary from farm to farm as the actual nutritional value of a diet will be affected by different factors related to the diet (feed manufacturing, physiochemical characteristics of feed ingredients, feed form and delivery method, etc.) [2,3,4], the management and housing conditions [5,6,7], and the animal (genetic, gender, weight, health status) [2,8,9], among others. Thus, fast analysis methods to assess feed digestibility at farm level are of great interest to reduce the high costs and time associated with traditional in vivo digestibility trials for nutrients and energy [10] and improve production efficiency and farm sustainability.

Near-infrared reflectance spectroscopy (NIRS) is widely used to predict the nutritional value of raw materials and complete feeds in feed mills to monitor product quality [10,11]. The success of this technique relies on its non-destructive and fast analysis of samples, the low cost per sample with little or no sample preparation, the lack of need to use chemicals, and the possibility of analysing samples at different places [12]. Thus, the same sample can be analysed several times, and a high number of samples can be analysed every day. 

Recent research has focused on assessing feed digestibility using faeces analysed via NIRS in different animal species [13,14,15]. Faeces contain information about the digestive process itself and are an easy material to collect at farm level. Bastianelli et al. [10,13] showed in chickens and pigs [16] that the use of faecal NIRS (FNIRS) can provide useful information as it accounts for digestibility due to animal factors with acceptable accuracy to be used for large-scale evaluations of digestibility trials. Moreover, the FNIRS technique can predict the chemical composition of diet and faeces as well as the apparent total tract digestibility (ATTD) coefficients with moderate accuracy [17,18,19], making it feasible for use in pig nutrition research where controlled digestibility trials are not possible, and for animal breeding programmes. Thus, FNIRS is a cost-effective and promising tool for measuring feed efficiency and digestibility [18]. 

All previous research conducted using the FNIRS technique has dried the faecal samples by drying or freeze-drying methods, followed by a grinding process to obtain a homogenic and low particle size faecal samples. However, no research has assessed the feasibility of using dried or freeze-dried faecal samples without grinding for FNIRS to assess chemical composition and ATTD coefficients. If results for ground and not ground samples were similar, we could avoid an important workload before analysis. Previous literature reported the feasibility of obtaining similar nutritional values from intact or ground raw feed material [20]. 

Therefore, we hypothesise that similar results in faeces chemical composition and ATTD coefficients will be obtained from analysing via NIRS freeze-dried not ground (FDNG) and freeze-dried ground (FDG) faecal samples. The objective of the present study was to compare the FNIRS technique using faecal samples in two forms, FDNG and FDG, to predict the faeces’ chemical composition and ATTD coefficients of nutrients.

## 2. Materials and Methods

### 2.1. Care and Use of Animals, Diets and Faecal Sampling

The study was conducted at the Teagasc Pig Research Facility in Fermoy, Co. Cork, Ireland, and received ethical approval from the Teagasc Animal Ethics Committee (TAEC 244/2019). Two batches of pigs were used in the present study. In both batches, 220 Danish Duroc × (Large White × Landrace) grower-finisher pigs born within one week were housed in mixed sex pens with a fully slatted concrete floor (2.4 × 4.2 m) containing a single wet-dry feeder (330 mm [width] × 370 mm [depth] × 1000 mm [height]; MA37, Verba, Netherlands) and one supplementary nipple drinker. Water and pelleted feed were provided ad libitum. Temperature was automatically controlled by a Big Dutchman 135 pro ventilation controller (Vechta, Germany), with water heating, air intake via a perforated ceiling, and mechanical exhaustion of stale air via a fan. The temperature in the finisher’s accommodation ranged from 17 to 21 °C. Artificial lighting (LED) was provided at a minimum light intensity level of no less than 40 Lux and typically averaging 140–160 Lux for a minimum continuous period of eight hours per day. Lighting was provided between 07:00 a.m. and 18:00 p.m. every day to coincide with natural daylight. Pens were enriched with a 1.20 m fixed larch wood post on one of the walls without impairing the available floor space.

The first batch of pigs (B1) was weighed per pen (*n* = 20 pens; 11 pigs/pen; 50.1 ± 3.44 kg body weight (BW) at 13 weeks of age. The second batch of pigs (B2) was weighed per pen (*n* = 20 pens; 11 pigs/pen; 87.0 ± 4.10 kg BW) at 18 weeks of age. For both B1 and B2, pens were assigned based on BW to five different dietary treatments and pigs were followed for 2 weeks. Diets were formulated to obtain a control diet [10.03 MJ of net energy (NE) or 13.40 MJ of metabolizable energy (ME), 160.0 g of crude protein (CP), and 9.5 g of standardized ileal digestive (SID) lysine (Lys) per kg of feed] that met or exceed the minimum nutrient requirements [21], and 4 suboptimal diets which were: low CP (10.03 MJ NE or 13.40 MJ ME, 132.0 g of CP, and 7.5 g of SID Lys per kg of feed), high CP (10.03 MJ NE or 13.40 MJ ME, 188.0 g of CP, and 11.5 g of SID Lys per kg of feed), low NE (9.61 MJ NE or 12.85 MJ ME, 160.0 g of CP, and 9.5 g of SID Lys per kg of feed) and high NE (10.45 MJ NE or 13.95 MJ ME; 160.0 g of CP, and 9.5 g of SID Lys per kg of feed). Ingredients and calculated and analysed nutrient diet composition is shown in Table 1. After a 10-day adaptation period, faecal samples were collected from the pen floor during the 6 following days in both B1 and B2. Each day, one faecal sample was collected from each pen (*n* = 20/day) adding up to a total of 120 faecal samples (*n* = 24/ treatment) from B1 at 15 weeks of age, and 120 faecal samples (*n* = 24/ treatment) from B2 at 20 weeks. Pigs went back to the common management of the Teagasc Pig Research Facility after the 15-day trial period. 

### 2.2. Feed Analysis

Feed samples of each diet were collected (duplicate) from the feeders and analysed for dry matter (DM), ash, CP, crude fibre, fat, total amino acid profile, and acid insoluble ash (AIA) at the Sciantec Analytical Services (Stockbridge Technology Centre, Cawood, Yorkshire, UK). Dry matter was determined by oven drying for 4 h at 103 °C [22]. Ash was determined via combustion in a muffle furnace at 550 °C [23]. Organic matter (OM) was calculated as *1000—Moisture—Ash*. Crude protein was determined as N × 6.25 based on the DUMAS method [24] using the LECO FP-628 analyser (Leco Instruments Ltd., Stockport, UK). Crude fibre was measured by a Fibertec semi-automatic system (Tecator, Höganäs, Sweden) using the gravimetric method [22]. Gross energy (GE) was determined using an adiabatic bomb calorimeter (Parr Instrument Company, Moline, IL, USA). Fat was determined using the Randall/Soxtec/Submersion method [25]. Amino acid determination was carried out based on the ion exchange high performance liquid chromatography technique [26] using the Biochrom Amino Acid Analyser Sodium System (Biochrom Ltd., Cambridge, UK). Acid-insoluble ash was determined according to McCarthy et al. [27]. The native AIA was used for the digestibility estimations. 

### 2.3. Faecal Analysis

Faecal samples were frozen at −20 °C after collection, then freeze-dried and ground using a FOSS Cyclotec 1093 Sample Mill (Foss, Denmark) with a 1 mm sieve. A total of 10 faecal samples had to be discarded because of a technical problem with the freeze-dryer machine. Faecal chemical analyses were conducted at Sciantec Analytical Services (Stockbridge Technology Centre, Cawood, Yorkshire, UK). Dry matter, ash, OM, CP, GE, fat, and AIA parameters were determined or calculated using the same methods previously described in the feed analysis section. 

### 2.4. Determination of Nutrient and Energy Digestibility

The chemical analyses of diets and faeces allowed for the determination of ATTD coefficients for all the analysed nutrients and energy. The ATTD of the nutrients was calculated using the following equation [28]:

$$ATTD\;coefficient=1-\left(\frac{Nutrient\;in\;faeces}{Nutrient\;in\;feed}\right)\times \left(\frac{AIA\;in\;feed}{AIA\;in\;faeces}\right)$$

### 2.5. Faecal NIRS Analysis

**Figure 1:**
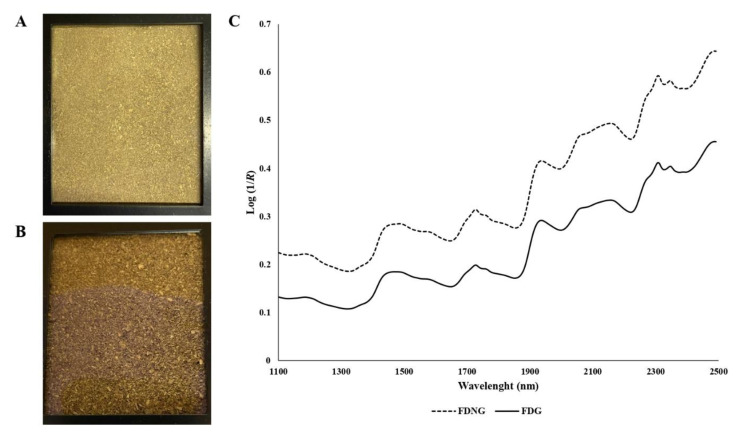
(**C**) Log (1/*R*) average spectra of freeze-dried not ground faeces (FDNG; (**B**)) and freeze-dried faeces (FDG; (**A**)) analysed using NIRS from growing and finishing pigs at 13 and 18 weeks of age, batch 1 and batch 2, respectively.

Faecal NIRS spectra were obtained from the same samples that were used for chemical analysis. Faecal samples were scanned via NIRS two times, first as FDNG and then as FDG. Faecal samples were scanned on a FOSS monochromatic spectrometer, NIRSystem 6500 (Foss NIRSystems, Hillerød, Denmark), in reflectance mode from 1100 to 2498 nm (with 2 nm steps). The analysis of FDNG and FDG faecal samples was carried out using the 1/4 rectangular cup transport cell that was 4.6 cm wide and 5.7 cm long. Two replicates were measured for each sample, using the average of the spectra for calibration. Spectral absorbance values were obtained as log (1/*R*), where *R* is sample reflectance. Spectra data were collected using the WinISI software package (version 4.10.0, Infrasoft International LLC, State College, PA, USA). Log (1/*R*) average spectra of FDG and FDNG are shown in Figure 1. 

### 2.6. Development of Faecal NIRS Calibration Equations and Statistical Analysis

Prior to the calibration procedures, spectral data was subjected to an analysis of its structure and variability in the sample population using the CENTER algorithm included in the WinISI software package. Thus, a principal component analysis and calculation of the Mahalanobis distance (GH) were performed. The latter calculates the distance of each spectrum sample from the center of the population in an n-dimensional space. Then, samples with a statistical value of more than 3 GH were considered outliers or anomalous spectra [29]. For this analysis, standard normal variate (SNV) and detrending (DT) methods were used for scatter correction [30]. Moreover, a first-derivative treatment of “1,5,5,1” was used. The first digit is the derivative number, the second digit is the gap over which the derivative is calculated, the third digit is the number of data points in a moving average, or first smoothing, and the fourth digit is the second smoothing [31]. During this process, a total of seven samples were deleted from both the FDNG and FDG data sets so 223 faecal samples were finally available for the calibration procedures. Descriptive statistics of the final calibration data set are provided in Table 2. Descriptive statistics were conducted using the software SAS v9.4 (SAS Institute Inc., Cary, NC, USA). 

The calibration procedure was performed using the modified partial least squares regression method. Using this method, the NIR residuals at each wavelength are standardised before calculating the next factor [29]. Then, FNIRS calibration was performed to predict DM, CP, OM, GE, fat, and the ATTD for DM, CP, OM, GE, and fat, based on the FDG and FDNG faeces spectra. The objective of the study was to compare the FDG versus the FDNG calibrations on pig faeces; thus, all data was used to perform the calibration and was not divided into calibration and validation data sets. Calibrations were performed based on cross-validation methods. Scatter correction was applied to all calibrations using SNV and DT [30]. The SNV approach is used to remove multiplicative interferences of scatter and particle size, while DT is used to remove variations in the baseline shift and curvilinearity that are usually found in the reflectance spectra [30]. Moreover, a total of eight derivative mathematical treatments were tested for the calibration procedure: 1,4,4,1; 1,8,4,1; 1,5,5,1; 1,10,5,1; 2,4,4,1; 2,8,4,1; 2,5,5,1; and 2,10,5,1. 

For both FDG and FDGN calibration data sets, cross-validation was performed using two different methods. The first method consisted of randomly splitting the data into four equal cross-validation groups. The second method was leave-one-out cross-validation. The number of terms was limited to 15 for the faeces calibrations [17]. The best calibration equations were selected according to the standard error of calibration (SEC), the coefficient of determination for calibration (R^2^_c_), the standard error of cross-validation (SECV), and the coefficient of determination for cross-validation (R^2^_cv_). Moreover, the residual predictive deviation (RPD) was calculated to describe the accuracy of the calibration equations and was calculated as the ratio of the standard deviation of the reference data to the SECV [32]. 

Finally, the best and final selected calibration equations for each faeces chemical and ATTD components for FDNG and FDG were compared using the Fisher’s Test [33,34]. *F* value is calculated as:

$$F=\frac{{{(SECV}_{2})}^{2}}{{{(SECV}_{1})}^{2}}$$

where SECV_1_ and SECV_2_ are from two different models (FDNG and FDG) and SECV_1_ < SECV_2_. Then, *F* is compared to the *F*_critical_ (1—P, *n*_1_—1, *n*_2_—1) with *p* = 0.05 and *n*—1 degrees of freedom. The *F*_critical_ can be obtained in the *F* table, where *P* is the significance level, *n*_1_ is the number of times that the measure is repeated in the FDNG model, and *n*_2_ is the number of times that the measure is repeated in the FDG model. Differences between the SECV values are significant when *F* > *F*_critical_.

## 3. Results

### 3.1. Faeces Chemical and ATTD Nutrient Composition

Descriptive statistics for each experimental diet for the faeces’ chemical components and ATTD nutrient composition are provided in Table 2. Diets were formulated to generate an important variation in the calibration data set for both chemical components of faeces and ATTD of nutrients. A wide range in nutrient composition was observed, especially for CP (218.0–320.0 g/kg) and fat (32.0–79.0 g/kg) for faeces chemical components, and all ATTD nutrients. However, SD was low for most parameters, and the highest coefficients of variation were observed for CP (9.0% and 9.1%) and fat (15.7% and 7.9%) for both chemical and ATTD components, respectively.

### 3.2. Faecal NIRS Calibrations

Faecal NIRS calibration equations were successfully developed for all parameters to predict the faeces’ chemical composition and ATTD coefficients (Table 3). Similar results were obtained using both cross-validations with 4 random groups and leave-one-out methods. Therefore, only leave-one-out cross-validation results will be further discussed. Overall, faeces’ chemical components were better predicted than ATTD coefficients in both FDG and FDNG faeces. 

Chemical components such as DM and CP were successfully predicted with R^2^_cv_ close to 0.9 and RPD values close to or higher than 3 for both FDG and FDNG faeces. However, predictions of OM and GE were less accurate, with R^2^_cv_ values between 0.7 and 0.8 and RPD values closer to 2. Lastly, fat had the least accurate prediction, with R^2^_cv_ values close to 0.6 and RPD values lower than 2. 

The ATTD coefficients had moderate prediction accuracy, with R^2^_cv_ values ranging from 0.54 to 0.67 and RPD values lower than 2 in FDG faeces, while R^2^_cv_ values ranged between 0.60 and 0.75 and DM and OM had RPD values higher than 2 in FDNG faeces.

### 3.3. Freeze-Dried Not Ground vs. Freeze-Dried Ground Models

Results from the Fisher’s Test to differentiate FDG and FDNG models for the different faeces chemical and ATTD parameters are reported in Table 4. Chemical component calibration equations for DM, OM, GE, and fat were not different between FDG and FDNG (*p* > 0.05), while CP differed between FDG and FDNG (*p* < 0.05) with SECV being lower in the FDG model. The ATTD coefficient calibration equations for CP, GE, and fat were not different between FDG and FDNG (*p* > 0.05), although SECV was numerically lower in CP and GE values of the FDNG compared to FDG. The ATTD DM and OM coefficients differed between FDG and FDNG (*p* < 0.05) with SECV being lower in the FDNG model in both cases.

## 4. Discussion

The results from the present study show that it is possible to successfully predict with good accuracy the chemical components of faeces and with moderate accuracy the ATTD coefficients by using FNIRS. Furthermore, similar prediction equations can be obtained by using FDG and FDNG faeces via NIRS. It is worth mentioning that the use of FNIRS is not novel since several studies have been conducted in cattle [15,35,36], poultry [10,13], or rabbits [14,37], among others. Bastianelli et al. [16], Schiborra et al. [19], Nirea et al. [18], and Paternostre et al. [17] are the only studies on the use of FNIRS in pigs that appear in the literature. These studies may be compared with the present study by using the RPD value. The latter allows SECV to be standardised and compare the results obtained by previous reports that used different data (means, standard deviations, ranges, etc.) obtained in different conditions from the data used in the present study [32]. Williams [32] established a RPD value to be acceptable when it is above 3.0, although Chang et al. [38] suggested good accuracy when RPD > 2.0, moderate accuracy when RPD ranges between 1.4 and 2.0, and poor accuracy when RPD < 1.4. Minasny and McBratney [39] suggested that a good calibration equation and RPD value are subject to the author’s interpretation. Moreover, the RPD value will be influenced by the kind of sample, its preparation, and how it is presented to the NIRS instrument, the variance observed in the data set used for calibration, and the possible error of the reference method [40]. Additionally, Shenk et al. [41] suggested an excellent calibration when R^2^_cv_ ≥ 0.90, a good calibration when R^2^_cv_ = 0.70–0.89, while a calibration with a R^2^_cv_ = 0.50–0.69 could establish a classification with a good separation between the high, medium, and low values of the parameters being analysed. In the present study, we consider a RPD value above 3 with a R^2^_cv_ ≥ 0.90 an excellent accuracy calibration, a RPD value between 2.0 and 3.0 with a R^2^_cv_ = 0.70–0.89 a good accuracy calibration, a RPD value between 1.5 and 2.0 with a R^2^_cv_ = 0.50–0.69 a moderate accuracy calibration, and a RPD value below 1.5 with a R^2^_cv_ = 0.50 a poor accuracy calibration. 

Faecal CP showed excellent accuracy with RPD values above 3.0 and R^2^_cv_ > 0.90 in the present study. This outcome is in agreement with previous studies using pigs, which found RPD and R^2^_cv_ values close to 3.0 and 0.90, respectively [17,18]. Differences with other authors who obtained lower RPD and R^2^_cv_ values in CP compared to the present study [16] could be related to a low SD of the reference values of the calibration set, as RPD and R^2^_cv_ are dependent on the range of values [42]. Bastianelli et al. [16] assessed feed digestibility using FNIRS, accounting for animal factors, but fed with the same diet. The latter might explain the low SD of their calibration data set, but at the same time, the potential use of FNIRS for animal genetic digestibility trials as an example. In agreement with previous studies [17,19], the accuracy for DM was excellent, while for OM it was good. Gross energy calibrations were good with RPD values close to 2.0 and R^2^_cv_ values between 0.70 and 0.75, similar to those obtained by Paternostre et al. [17]. Nirea et al. [18] obtained higher values of RPD and R^2^_cv_ for OM and GE that are explained by the range of their calibration data set [42], which also resulted in a higher SECV. Fat calibrations had a moderate accuracy similar to previous literature [18], which could be enough to distinguish between high, medium, and low levels in faecal samples. 

Calibrations for ATTD coefficients of nutrients had a moderate accuracy with RPD values between 1.5 and 2.0 and R^2^_cv_ between 0.55 and 0.75 in the present study. These findings are similar to previous studies in pigs [16,18,19] and differences between them may depend on the range and variability of the reference data used for calibration [42]. The digestibility results obtained in the present study could be improved by combining the faeces and feed spectra, as has been previously demonstrated in ruminants [43], poultry [44], and recently in pigs [17]. Nevertheless, depending on the objective of the calibrations, a moderate accuracy could usefully distinguish between high, medium, and low levels of ATTD coefficients of nutrients in faecal samples, which, in practical conditions, could serve as a tool for early detection of digestive problems and/or to improve performance. Moreover, further research could focus on building a prediction equation for ATTD coefficient of nutrients based on the estimated chemical composition of feed and faeces by the use of NIRS, considering that calibrations for chemical composition of faeces were more accurate than calibrations for the ATTD coefficient of nutrients. Additionally, further research could explore the correlation between high, medium, and low ATTD coefficients of nutrients with factors such as farm management, feeding management, feed ingredients, farm facilities, health status, environment, and season, among others. 

Overall, the present study reaffirms FNIRS as a potential tool to evaluate faeces chemical components and ATTD coefficients of nutrients at farm level by collecting faeces from the pen floor. Further studies might explore the possibility of differentiating suboptimal diets in protein and energy levels by using FNIRS and establish the level of accuracy needed for the calibration equations to differentiate, for instance, when the animals are fed high levels of protein above their nutrient requirements at farm level. 

A limitation of the present study was the absence of a complete external validation data set to corroborate the robustness of the calibrations. Some previous studies using FNIRS in pigs conducted internal validations to assess the quality and robustness of the calibration equations [16,18] using a subset of the total data set, not used for the calibration process. However, no previous study has assessed the quality and robustness of the calibration equations by using a complete external validation data set. In the present study, the robustness of the calibrations was assessed by using two cross-validation methods: (four random groups and leave-one-out). In both cases, the SECV obtained for each parameter was similar. A further study could assess the validation of a calibration predicting faeces chemical components and ATTD coefficients of nutrients by using a complete external validation data set to understand how accurate the calibration is and quantify how many faecal samples are needed to obtain a robust calibration feasible to be implemented in practical conditions. Nevertheless, it is important to consider that FNIRS calibrations should be revised regularly in practical conditions, adding new information to obtain a wider range of data sets, and making better predictions when information comes from other environments (different feed, sex, genetic, health, farm, etc.). 

The present study compared the faeces chemical and ATTD coefficients from analysing via FNIRS faecal samples in FDNG and FDG forms. The concern was that the difference in particle size between FDNG and FDG faecal samples could cause a scatter effect in FDNG faecal samples due to a deviation of light from a straight trajectory into different paths [20]. Nonetheless, the pre-treatment of the raw spectra [45] by using SNV, DT, and derivative methods allows to reduce the differences observed in the raw spectra and obtain similar prediction results for both FDNG and FDG faecal samples. However, differences in some parameters were observed when comparing the prediction equations of FDNG and FDG. In any case, the magnitude of the differences and the possible loss of precision and accuracy seem minor compared to the advantages obtained by using FDNG faecal samples in FNIRS, which are faster analysis while reducing the workload associated with the faecal grounding process. Further research should explore the possibility of predicting faeces chemical components and ATTD coefficients of nutrients by using the FNIRS technique with fresh faecal samples, which would be an important step towards facilitating the sample procedure and analysis while reducing the workload and investment and allowing early detection of health problems related to the digestion process. With the appearance and advances of different types of NIRS instruments adapted to different circumstances [46], one NIRS instrument could be able to analyse these fresh faeces and even do it at the farm level and not in the laboratory. Finally, further research could explore the use of NIRS as a potential tool for health monitoring to understand how precise it could be in identifying sick vs. non-sick animals based on specificity and sensitivity. 

## 5. Conclusions

This study has shown that faeces chemical components and ATTD coefficients of nutrients are successfully predicted using FNIRS with freeze-dried ground or not ground faecal samples. The latter outcome facilitates the FNIRS analysis being faster with less workload because it avoids the grinding process. Further research might explore the use of fresh faecal samples analysed via NIRS. Moreover, the present study reaffirms the FNIRS as a potential tool to evaluate faeces’ chemical components and ATTD coefficients of nutrients.

## Figures and Tables

**Table 1:** Ingredient, calculated, and analysed nutrient composition on an as-fed basis of the five dietary treatments ^1^.

	Diets ^2^				
	Control	LCP	HCP	LNE	HNE
Ingredients, g/kg					
Wheat	350.0	350.0	350.0	330.0	306.2
Barley	282.5	345.0	0.0	310.5	200.0
Maize	150.0	150.0	286.6	100.0	275.5
Soybean meal 47.5	172.4	95.7	254.1	175.1	172.4
Soybean hulls	14.2	29.7	63.9	58.3	0.0
Vegetable Oil	5.0	5.0	17.6	0.0	21.5
Calcium carbonate	12.3	12.7	12.2	10.7	11.7
Dicalcium phosphate anhydrous	0.50	0.50	1.00	3.00	0.50
Sodium chloride	4.50	4.40	3.20	4.40	3.70
L-Lysine HCl	4.30	3.80	5.30	4.15	4.40
L-Threonine	1.60	1.20	2.20	1.15	1.60
DL-Methionine	1.30	0.70	2.20	1.30	1.20
L-Tryptophan	0.20	0.10	0.20	0.20	0.10
L-Valine	0.00	0.00	0.30	0.00	0.00
Vitamin and trace mineral mixture ^3^	1.20	1.20	1.20	1.20	1.20
Calculated/Analysed Composition ^4^, % as fed or as specified					
Dry Matter, analysed	88.00	87.70	88.30	87.90	87.90
Ash, analysed	3.90	3.60	4.00	4.10	3.90
ME, MJ/kg	13.40	13.40	13.40	12.85	13.95
NE, MJ/kg	10.03	10.03	10.03	9.61	10.45
SID Lys:NE, g/MJ	0.95	0.75	1.15	0.99	0.91
Crude Protein, analysed	13.40	11.60	16.20	14.50	14.30
Total Lys, analysed	1.05	0.88	1.31	1.08	1.02
Total Thr/Lys ratio, analysed	0.58	0.58	0.56	0.57	0.62
Total Met-Cys/Lys ratio, analysed	0.64	0.64	0.63	0.65	0.68
Total Trp/Lys ratio, analysed	0.14	0.15	0.15	0.14	0.14
Total Val/Lys ratio, analysed	0.60	0.65	0.65	0.67	0.69
Total Leu/Lys ratio, analysed	1.09	1.14	1.08	1.14	1.14
Total Ile/Lys ratio, analysed	0.54	0.55	0.57	0.58	0.60
Total His/Lys ratio, analysed	0.34	0.38	0.37	0.36	0.38
SID Lys	0.95	0.75	1.15	0.95	0.95
SID Thr/Lys ratio	0.65	0.65	0.65	0.65	0.65
SID Met-Cys/Lys ratio	0.59	0.59	0.59	0.59	0.59
SID Trp/Lys ratio	0.19	0.19	0.19	0.19	0.19
SID Val/Lys ratio	0.66	0.67	0.65	0.66	0.66
SID Leu/Lys ratio	1.16	1.20	1.11	1.16	1.15
SID Ile/Lys ratio	0.56	0.55	0.57	0.57	0.57
SID His/Lys ratio	0.36	0.36	0.34	0.35	0.36
Fat, analysed	2.79	2.74	3.78	2.21	4.19
Crude Fibre, analysed	2.90	3.40	4.20	4.20	2.40
NDF	12.96	14.15	13.54	15.02	12.00
Calcium	0.75	0.75	0.80	0.77	0.72
Digestible Phosphorus	0.22	0.22	0.22	0.25	0.22

^1^ Diets were fed to growing and finishing pigs for 15 days at 13 and 18 weeks of age, batch 1 and batch 2 respectively. ^2^ LCP = Low Crude Protein; HCP = High Crude Protein; LNE = Low Net Energy; HNE = High Net Energy. ^3^ Provided per each kg of feed: 60 mg Copper sulphate, 80 mg Ferrous sulphate monohydrate, 50 mg Manganese oxide, 100 mg Zinc oxide, 0.5 mg Potassium iodate, 0.4 mg Sodium selenite, 2 MIU Vitamin A, 0.5 MIU Vitamin D_3_, 40 MIU Vitamin E, 4 mg Vitamin K, 0.015 mg Vitamin B_12_, 2 mg Riboflavin, 12 mg Nicotinic acid, 10 mg Pantothenic acid, 2 mg Vitamin B_1_, 3 mg Vitamin B_6_. ^4^ ME = Metabolizable Energy; NE = Net Energy; SID = Standardized Ileal Digestible; NDF = Neutral Detergent Fibre.

**Table 2:** Descriptive statistics of faeces chemical composition and apparent total-tract digestibility (ATTD) of nutrients for the final calibration data set from each experimental diet fed to growing and finishing pigs at 13 and 18 weeks of age, batch 1 and batch 2, respectively ^1,2^.

Constituent ^3^	Chemical Analysis					ATTD				
	Mean	SD	Min	Max	CV	Mean	SD	Min	Max	CV
Total data set, *n* = 223										
DM, g/kg	930.0	16.40	901.0	957.0	0.018	0.83	0.038	0.71	0.90	0.046
CP/DM, g/kg	266.0	23.90	218.0	320.0	0.090	0.71	0.065	0.52	0.83	0.091
OM/DM, g/kg	971.0	14.50	940.0	1012.0	0.015	0.85	0.035	0.74	0.91	0.041
GE/DM, MJ/Kg	20.3	0.38	19.1	21.3	0.019	0.79	0.045	0.63	0.88	0.056
FAT/DM, g/kg	47.0	7.30	32.0	79.0	0.157	0.77	0.061	0.62	0.90	0.079
Control diet, *n* = 46										
DM, g/kg	929.0	17.00	903.0	951.0	0.018	0.87	0.016	0.82	0.90	0.019
CP/DM, g/kg	258.0	21.00	221.0	304.0	0.081	0.77	0.029	0.72	0.82	0.037
OM/DM, g/kg	975.0	16.00	944.0	1005.0	0.016	0.88	0.016	0.84	0.91	0.018
GE/DM, MJ/Kg	20.3	0.22	19.9	20.8	0.011	0.83	0.020	0.77	0.87	0.024
FAT/DM, g/kg	45.0	5.30	36.0	61.0	0.118	0.81	0.034	0.74	0.88	0.042
LCP diet, *n* = 47										
DM, g/kg	931.0	16.10	911.0	955.0	0.017	0.82	0.029	0.73	0.88	0.036
CP/DM, g/kg	246.0	12.20	218.0	268.0	0.050	0.66	0.053	0.52	0.78	0.080
OM/DM, g/kg	975.0	15.10	946.0	1012.0	0.015	0.84	0.027	0.76	0.90	0.032
GE/DM, MJ/Kg	20.3	0.26	19.7	20.8	0.013	0.75	0.038	0.63	0.84	0.051
FAT/DM, g/kg	46.0	6.80	32.0	67.0	0.146	0.73	0.051	0.62	0.82	0.071
HCP diet, *n* = 41										
DM, g/kg	928.0	17.50	901.0	955.0	0.019	0.79	0.042	0.71	0.88	0.053
CP/DM, g/kg	298.0	13.30	262.0	320.0	0.045	0.66	0.062	0.55	0.80	0.094
OM/DM, g/kg	967.0	11.60	940.0	992.0	0.012	0.81	0.039	0.74	0.89	0.047
GE/DM, MJ/Kg	20.2	0.37	19.3	21.1	0.018	0.76	0.045	0.68	0.86	0.059
FAT/DM, g/kg	48.0	6.70	37.0	69.0	0.140	0.77	0.061	0.62	0.86	0.080
LNE diet, *n* = 44										
DM, g/kg	935.0	15.50	909.0	957.0	0.017	0.84	0.022	0.80	0.90	0.027
CP/DM, g/kg	271.0	14.20	243.0	310.0	0.052	0.73	0.035	0.67	0.83	0.048
OM/DM, g/kg	965.0	11.70	945.0	997.0	0.012	0.86	0.020	0.82	0.91	0.024
GE/DM, MJ/Kg	20.0	0.34	19.1	21.1	0.017	0.80	0.026	0.76	0.88	0.032
FAT/DM, g/kg	41.0	5.30	32.0	55.0	0.129	0.73	0.054	0.62	0.87	0.073
HNE diet, *n* = 45										
DM, g/kg	929.0	15.20	904.0	952.0	0.016	0.83	0.032	0.75	0.90	0.039
CP/DM, g/kg	263.0	20.40	225.0	300.0	0.078	0.72	0.051	0.59	0.83	0.071
OM/DM, g/kg	975.0	13.70	949.0	1010.0	0.014	0.85	0.030	0.77	0.91	0.035
GE/DM, MJ/Kg	20.7	0.39	19.7	21.3	0.019	0.80	0.036	0.71	0.88	0.045
FAT/DM, g/kg	53.0	6.80	41.0	79.0	0.129	0.81	0.045	0.67	0.90	0.056

^1^ Descriptive statistics were conducted using the software SAS v9.4 (SAS Institute Inc., Cary, NC, USA). ^2^ SD = Standard Deviation; Min = Minimum; Max = Maximum; CV = Coefficient of Variation. ^3^ LCP = Low Crude Protein; HCP = High Crude Protein; LNE = Low Net Energy; HNE = High Net Energy; DM = Dry Matter; CP = Crude Protein; OM = Organic Matter; GE = Gross Energy.

**Table 3:** Faecal NIRS calibration statistics of the selected calibration equations to predict the faeces chemical composition and the apparent total tract digestibility coefficients of grower-finisher pigs using freeze-dried ground and not ground faeces ^1,2^.

Constituent ^3^	Mean_cv4_	SD_cv4_	SEC_cv4_	R^2^c_cv4_	SECV_cv4_	R^2^cv_cv4_	RPD_cv4_	Mean_lo_	SD_lo_	SEC_lo_	R^2^c_lo_	SECV_lo_	R^2^cv_lo_	RPD_lo_
Freeze-dried ground faeces														
Faeces chemical components														
DM, g/kg	930.0	16.50	5.00	0.91	5.50	0.89	2.98	930.0	16.50	4.60	0.92	5.40	0.89	3.02
CP/DM, g/kg	266.0	23.80	4.80	0.96	5.60	0.95	4.31	267.0	23.90	5.10	0.96	5.40	0.95	4.45
OM/DM, g/kg	971.0	13.70	5.90	0.81	6.70	0.76	2.17	971.0	14.20	6.10	0.82	6.70	0.77	2.15
GE/DM, MJ/kg	20.3	0.36	0.16	0.80	0.18	0.75	2.18	20.3	0.36	0.16	0.79	0.18	0.74	2.11
FAT/DM, g/kg	46.0	6.10	3.40	0.69	3.80	0.62	1.95	46.0	6.10	3.60	0.66	3.80	0.61	1.91
Apparent total tract of nutrient digestibility														
dDM	0.83	0.034	0.017	0.75	0.019	0.70	2.05	0.83	0.036	0.020	0.71	0.022	0.64	1.75
dCP	0.71	0.062	0.037	0.63	0.040	0.58	1.62	0.71	0.061	0.036	0.65	0.041	0.54	1.57
dOM	0.85	0.032	0.016	0.75	0.017	0.71	2.04	0.85	0.031	0.016	0.73	0.018	0.67	1.98
dGE	0.79	0.041	0.024	0.66	0.026	0.59	1.70	0.79	0.039	0.022	0.69	0.024	0.62	1.85
dFAT	0.77	0.058	0.028	0.77	0.033	0.68	1.87	0.77	0.058	0.028	0.77	0.033	0.67	1.83
Freeze-dried not ground faeces														
Faeces chemical components														
DM, g/kg	931.0	16.20	4.90	0.91	5.70	0.88	2.85	930.0	16.30	5.00	0.91	5.70	0.88	2.86
CP/DM, g/kg	266.0	23.40	6.10	0.93	6.40	0.93	3.77	266.0	23.60	6.10	0.93	6.60	0.92	3.62
OM/DM, g/kg	971.0	14.00	6.60	0.78	7.30	0.73	2.00	971.0	14.20	6.40	0.80	7.40	0.73	1.96
GE/DM, MJ/kg	20.3	0.35	0.17	0.77	0.18	0.74	2.14	20.3	0.36	0.19	0.73	0.20	0.69	1.92
FAT/DM, g/kg	46.0	6.20	3.40	0.70	3.70	0.64	1.97	46.0	6.50	4.00	0.63	4.20	0.57	1.73
Apparent total tract of nutrient digestibility														
dDM	0.83	0.036	0.017	0.76	0.019	0.70	1.97	0.83	0.035	0.015	0.81	0.018	0.73	2.09
dCP	0.71	0.058	0.030	0.73	0.034	0.66	1.91	0.71	0.062	0.035	0.68	0.039	0.61	1.67
dOM	0.85	0.033	0.015	0.78	0.017	0.72	1.99	0.85	0.032	0.013	0.83	0.016	0.75	2.21
dGE	0.79	0.039	0.019	0.76	0.023	0.65	1.96	0.79	0.039	0.019	0.77	0.023	0.67	1.96
dFAT	0.77	0.059	0.029	0.76	0.034	0.67	1.83	0.77	0.059	0.028	0.78	0.033	0.67	1.85

^1^ Two different batches of pigs were used, one batch (50.1 ± 3.44 kg body weight) was used at 13 weeks of age and the second batch (87.0 ± 4.10 kg body weight) was used at 18 weeks of age. ^2^ Faecal NIRS calibrations were performed based on two cross-validation methods: Cross-validation in groups of 4 (CV4) and leave-one-out cross-validations (Lo). SD = Standard Deviation; SEC = Standard Error Calibration; R^2^_c_ = Coefficient of Determination for Calibration; SECV = Standard Error Cross-Validation; R^2^_cv_ = Coefficient of Determination for Cross-Validation; RPD = Residual Predictive Deviation. ^3^ DM = Dry Matter; CP = Crude Protein; OM = Organic Matter; GE = Gross Energy; dDM = digestible Dry Matter; dCP = digestible Crude Protein; dOM = digestible Organic Matter; dGE = digestible Gross Energy; dFAT = digestible Fat.

**Table 4:** Fisher’s test statistical comparison (*p* ≤ 0.05) between the leave-one-out standard error cross-validation (SECV_lo_) values obtained for the best models for predicting faeces chemical and apparent total tract digestibility parameters using freeze-dried not ground (FDNG) and freeze-dried ground (FDG) faecal samples ^1,2^.

Constituent ^3^	SECV_lo_		F	F_critical_
	FDG	FDNG		
Faeces chemical components				
DM, g/kg	5.40	5.70	1.10	1.25
CP/DM, g/kg	5.40	6.60	1.51	1.25
OM/DM, g/kg	6.70	7.40	1.20	1.25
GE/DM, MJ/kg	1.80	2.00	1.19	1.25
FAT/DM, g/kg	3.80	4.20	1.21	1.25
Apparent total tract of nutrient digestibility				
dDM	0.022	0.018	1.43	1.25
dCP	0.041	0.039	1.14	1.25
dOM	0.018	0.016	1.26	1.25
dGE	0.024	0.023	1.13	1.25
dFAT	0.033	0.033	1.01	1.25

^1^ Two different batches of pigs were used, one batch (50.1 ± 3.44 kg body weight) was used at 13 weeks of age and the second batch (87.0 ± 4.10 kg body weight) was used at 18 weeks of age. ^2^ Differences between the SECV_lo_ values are significant when *F* > *F*_critical_. Low SECV_lo_ values improve the quality of the calibration equations. ^3^ DM = Dry Matter; CP = Crude Protein; OM = Organic Matter; GE = Gross Energy; dDM = digestible Dry Matter; dCP = digestible Crude Protein; dOM = digestible Organic Matter; dGE = digestible Gross Energy; dFAT = digestible Fat.

## Data Availability

The datasets used and analysed during the current study are available from the corresponding author on reasonable request.

## Abbreviations

AIA: acid insoluble ash; ATTD: apparent total tract digestibility; B1: first batch; B2: second batch; BW: body weight; CP: crude protein; DM: dry matter; DT: detrending; FDG: freeze-dried ground; FDNG: freeze-dried not ground; FNIRS: faecal near-infrared reflectance spectroscopy; GE: gross energy; GH: Mahalanobis distance; Lys: lysine; ME: Metabolizable energy; NE: net energy; NIRS: near-infrared reflectance spectroscopy; OM: organic matter; R^2^_c_: coefficient of determination for calibration; R^2^_cv_: coefficient of determination for cross-validation; RPD: residual predictive deviation; SEC: standard error of calibration; SECV: standard error of cross-validation; SID: standardized ileal digestive; SNV: standard normal variate.
